# Prospective Effects of Self-Rated Health on Dementia Risk in Two Twin Studies of Aging

**DOI:** 10.1007/s10519-024-10182-1

**Published:** 2024-06-01

**Authors:** Matthew J. D. Pilgrim, Christopher R. Beam, Marianne Nygaard, Deborah Finkel

**Affiliations:** 1 Department of Psychology, University of Southern California, Los Angeles, USA; 2 Davis School of Gerontology, Universitty of Southern California, Los Angeles, USA; 3 The Danish Twin Registry, Department of Public Health, University of Southern Denmark, Odense, Denmark; 4 Center for Economic and Social Research, University of Southern California, Los Angeles, USA; 5 Institute for Gerontology, School of Health and Welfare, Jönköping University, Jönköping, Sweden

**Keywords:** Self-rated health, Dementia risk, Subjective health, Twins, Genetic risk

## Abstract

Subjective health ratings are associated with dementia risk such that those who rate their health more poorly have increased risk for dementia. The genetic and environmental mechanisms underlying this association are unclear, as prior research cannot rule out whether the association is due to genetic confounds. The current study addresses this gap in two samples of twins, one from Sweden (*N* = 548) and one from Denmark (*N* = 4,373). Using genetically-informed, bivariate regression models, we assessed whether additive genetic effects explained the association between subjective health and dementia risk as indexed by a latent variable proxy measure. Age at intake, sex, education, depressive symptomatology, and follow-up time between subjective health and dementia risk assessments were included as covariates. Results indicate that genetic variance and other sources of confounding accounted for the majority of the effect of subjective health ratings on dementia risk. After adjusting for genetic confounding and other covariates, a small correlation was observed between subjective health and latent dementia risk in the Danish sample (*r*_E_ = − .09, *p* < .05). The results provide further support for the genetic association between subjective health and dementia risk, and also suggest that subjective ratings of health measures may be useful for predicting dementia risk.

**Supplementary Information:**

The online version contains supplementary material available at 10.1007/s10519-024-10182-1.

Dementia is a worldwide health problem that continues to worsen as the proportion of older adults increases (Prince et al. 2016). It is a complex disorder with multiple etiological features (Raz et al. 2016) and a growing range of immutable and modifiable risk factors (Livingston et al. 2020). Among the set of modifiable risk variables, physical health factors including obesity, especially in midlife (Albanese et al. 2017; Xu et al. 2011), cardiovascular disease (Eriksson et al. 2010; Stefanidis et al. 2018), hypertension (McGrath et al. 2017), and diabetes (Xue et al. 2019) are considered to be alterable at most stages of the lifespan and are associated with multiple dementia etiologies (Bir et al. 2021; Profenno et al. 2010; Stampfer 2006). Subjective health ratings of overall health may encompass various modifiable risk factors of dementia but have been infrequently studied in this context (Stephan et al. 2021). The purpose of the current study is to extend prior research on the utility of self-rated health measures for predicting dementia risk. We used two samples of twins from Sweden and Denmark to test whether within-family differences in self-rated health predict dementia risk holding constant familial (i.e., genetic and shared environmental) confounds.

Subjective health ratings (hereafter referred to as “subjective health”) are self-rated measures of global health typically based on a single-item Likert scale. Although simple to administer in survey-based questionnaires, subjective health items have been linked to a variety of clinically relevant outcomes. For example, poorer subjective health is associated with increased mortality (Conde-Sala et al. 2020; DeSalvo et al. 2006; Idler & Benyamini 1997; Schnittker and Bacak 2014; Shen et al. 2014; Yu et al. 1998), chronic disease morbidity (Latham and Peek 2013), major cardiovascular events (Rutledge et al. 2010), stroke occurrence (Dong et al. 2018), depression (Ambresin et al. 2014), functional decline (Mccurry et al. 2002), serum inflammatory markers (Christian et al. 2011), and over fifty biomarkers from multiple organ systems (Kananen et al. 2021). Additionally, subjective health has been shown to reflect both physical aspects of health such as functional limitations (Krause and Jay 1994; Manderbacka 1998), number of chronic diseases (Meng and D’arcy 2016), diabetes and hypertension (Jorgensen et al., 2014), medications (Benyamini et al. 1999), and physically inactive life-style (Sargent-Cox et al. 2014), as well as psychological aspects of health such as depressive symptoms (Spuling et al. 2015) and personality traits (Stephan et al. 2020). Subjective health is, thus, an important predictor to study in the context of dementia as research indicates that it captures many aspects of individuals’ health status (e.g., Jylhä, 2009) and, therefore, provides a broader representation than any single objective health indicator.

Whereas subjective health has been operationalized in several ways, it often consists of individuals’ views of a constellation of interrelated health factors influenced by personal history, cultural factors, reference groups and expectations (Jylhä, 2009). However, the way in which subjective health is queried may change the health information it represents (Franz et al. 2017; Sargent-Cox et al. 2008). Most importantly, there are differences in which aspects of health predict individuals’ ratings of their global health vs. their health compared to their same-aged peers (Sargent-Cox et al. 2008). In addition, recent evidence suggests that the way in which individuals perceive their health to affect their activities may be predicted by different factors than their global subjective health (Finkel et al. 2020). In this way, various dimensions of subjective health may have unique characteristics, which is an important consideration for predictive investigations using subjective health measures.

Subjective health measures correlate with dementia risk (Ganguli et al. 2019; Hamid et al. 2010; Jia et al. 2020; John and Montgomery 2013; Lipnicki et al. 2019; Montlahuc et al. 2011; Sargent-Cox et al. 2011; Stephan et al. 2021; Weisen et al. 1999; Yip et al. 2006). Compared to individuals with subjective health ratings of “good”, those who rate their health as “poor” have an approximately fourfold increased risk of dementia (Hamid et al. 2010; Yip et al. 2006) and over twice the odds of mild cognitive impairment (Sargent-Cox et al. 2011). In addition, two longitudinal prospective studies with follow-up periods of over 5 years showed that those with poorer subjective health had 18–70% greater likelihood of incident dementia (Montlahuc et al. 2011; Stephan et al. 2021).

Despite studies showing an association between subjective health and dementia risk, the mechanism underlying their association is unclear. Poor subjective health may reflect other dementia risk factors such as hypertension, physical inactivity, and low social activity and it is variation in these underlying factors that account for variation in dementia (Stephan et al. 2021). Alternatively, subjective health may approximate aspects of an ongoing asymptomatic dementia process such as subjective memory complaints known to increase risk of eventual dementia diagnosis (Mitchell et al. 2014). One open question is whether genetic variance accounts for the association between poor physical health and dementia. For example, an Alzheimer’s disease polygenic score accounted for part of the observed association between subjective health and dementia diagnosis (Stephan et al. 2021). Yet, to date no quantitative genetic study has evaluated whether subjective health and dementia correlate because of shared genetic variance, unique environmental experiences, or both. Genetically informed (or “twin”) studies can help clarify whether and to what extent poor physical health predicts dementia risk by statistically adjusting for genetic and shared environmental sources of confounding that may fully or partially explain their association. Twin and sibling studies can be used to decompose variance and covariance into genetic and environmental components shared and unshared by members of the same family. As an example, consider monozygotic (MZ) pairs of twins in which one twin in each pair reports worse physical health than the co-twin. If these twins’ physical health continues to worsen, on average, and these twins also become more likely to show dementia symptoms later in life, genetic confounds cannot explain the association given that they are genetically matched with their physically healthier co-twins. In this way, twin studies in which random assignment to health status was not or could not be done are one methodology for ruling out a broad set of genetic and environmental confounds that may explain the association between physical health and dementia risk. We note, however, that like other observational studies, twin studies cannot account for other third variable confounds that may account for the association between physical health and dementia risk. For this reason, within-pair effects of physical health on dementia risk are regarded as “quasi-causal”, as these effects bolster the causal conclusions that can be made between physical health and dementia risk in lieu of random assignment.

In the present study, we use two population-based twin studies of aging to test effects of subjective health on dementia risk. To improve the generalizability of our findings, multiple framings of subjective health questions were used as variation in how individuals are asked to rate their health may affect what the measure captures (Finkel et al. 2020; Sargent-Cox et al. 2008). These measures include a general overall measure of physical health, a comparative measure of physical health, and a measure regarding the impact of health on everyday activities. Moreover, we used the genetically informative nature of the two studies to test whether effects of subjective health on dementia risk were statistically significant after adjusting for genetic and environmental confounds. Here, we also included demographic variables previously found to correlate with dementia risk, including age, sex, education, and depressive symptomatology, (Cherbuin et al. 2015; Livingston et al. 2020; Mazure and Swendsen 2016) as well as the follow-up time between subjective health and dementia risk measurements. We hypothesized that genetic confounds would account for a portion of the association between subjective health and dementia. We also hypothesized, however, that twins who reported worse health would have statistically significant greater risk of dementia, adjusting for the genetic and shared environmental correlation between subjective health measures and dementia risk.

## Method

### Sample

Data were drawn from two twin studies of aging: the Swedish Adoption Twin Study of Aging (SATSA; Finkel and Pedersen 2004) and the Longitudinal Study of Aging Danish Twins (LSADT; Christensen et al. 1999). Both samples include complete and incomplete pairs of monozygotic (MZ) and dizygotic (DZ) twins.

SATSA was initiated in 1984 using a subset of same-sex twins who were separated before the age of 10 and a demographically matched sample of twins reared together from the Swedish Twin Registry. Samples of twins reared apart and reared together were matched on sex, country of birth, and birth year. Twins in these subsamples did not statistically differ in terms of depressive symptomatology, subjective health outcomes, or latent dementia risk scores. We note, however, that there was a significant difference in education (*p* < .01) but that the difference was small (Cohen’s* d* = 0.23) between rearing subsamples. In the present study, we pooled twins reared apart (*N* = 283) and twins reared together (*N* = 265) for the reason that previous studies of subjective health have demonstrated that shared-environmental variance is negligible in older individuals (Franz et al. 2017) and rearing status rarely contributes to subjective or objective health measures (Harris et al. 1992). SATSA data collection concluded in 2014.

LSADT began in 1995 and included in-person interviews of all same-sex twins born in Denmark before 1920, who were therefore 75 years or older. Refreshment samples were added in 1997, 1999, and 2001 and included twins who were 70 years or older (Christensen et al. 2003; Pedersen et al. 2019). LSADT twins were interviewed every two years until 2005 when data collection in LSADT concluded.

In both SATSA and LSADT, we used twins’ earliest measures of subjective health and their final measure of cognitive and functional ability to maximize the time between measurements. Only twins who included at least one measurement of subjective health before their last cognitive testing were used in the study. Twins who did not provide cognitive data were excluded from both analytic samples. In SATSA, 548 individual twins (*N*_*MZpair*_ = 86, *N*_*MZsingletons*_ = 41, *N*_*DZpair*_ = 123, *N*_*DZsingletons*_ = 89) and 4,373 LSADT twins (*N*_*MZpair*_ = 443, *N*_*MZsingletons*_ = 669, *N*_*DZpair*_ = 646, *N*_*DZsingletons*_ = 1526) met the inclusion criteria. The mean age of the sample was 54.95 (*SD* = 10.02) for SATSA and 77.23 (*SD* = 5.34) for LSADT at SRH assessment. The mean follow-up age for SATSA was 77.77 (*SD* = 8.92) and 80.59 (*SD* = 6.31) for LSADT at cognitive assessment. Sixty percent and 59% of the twins were female in SATSA and LSADT, respectively.

### Measures

#### Subjective health

In SATSA, three separate measures of subject health were used to capture different aspects of how individuals perceived their own health (Gatz et al. 2015): a general self-rated health measure (SRH), a comparative health measure (COMP) and health impact on activities measure (ACT). SRH assessed, “How do you rate your general health status?” with three possible response options: 1 = “good,” 2 = “reasonable,” and 3 = “bad.” COMP assessed, “How do you rate your health status compared to others your age?” with three possible response options: 1 = “better,” 2 = “about the same,” and 3 = “worse.” ACT assessed, “Do you think your health prevents you from doing things you would like to do?” with three possible response options: 1 = “not at all,” 2 = “partly,” and 3 = “to a great extent.” LSADT contained a single subjective health item, SRH, worded as “How do you consider your present health in general?” with five possible response options: 1 = “excellent,” 2 = “good,” 3 = “acceptable,” 4 = “poor,” 5 = “very poor.” In order to put LSADT and SATSA on a common metric, each of the subjective health scores (SRH, COMP, and ACT in SATSA and SRH in LSADT) were converted to *T*-scores. *T*-scores were created by computing z-scores, multiplying them by ten, and then centering the scores around 50. Values were recoded such that higher scores indicate worse subjective health.

#### Latent dementia index

Dementia risk was quantified using latent dementia index (LDI) scores. SATSA and LSADT are part of a larger consortium of twin studies of aging: the Interplay of Genes and Environments Across Multiple Studies (IGEMS; Pedersen et al. 2013, 2019). As not all twins were assessed for dementia risk in SATSA and no twins were assessed and diagnosed directly in LSADT, LDI scores were constructed using a single-factor latent variable model that accounts for common variance in memory measures of cognitive ability, non-memory measures of cognitive ability, and functional ability variables taken from the in-person testing sessions of SATSA and LSADT. Specifically, in SATSA, the LDI included scores from immediate and delayed word list recall, delayed word list recognition, block design, forward and backward digit span, figure identification and rotation, letter fluency, information, synonyms, and decoding tests as well as informant rating of participant ability to complete daily tasks of living. In LSADT, LDI included immediate and delayed word list recall, forward and backward digit span, semantic fluency, and decoding tests as well as the sum of seven selected items from Lawton’s self-maintaining and instrumental activities measure (Lawton and Brody 1969). LDI approaches have been found to be reliable and valid, including in these two studies (Beam et al. 2022; Gavett et al. 2015; Royal et al., 2013). LDI scores were estimated using twins’ cognitive and functional ability data *after* their subjective health measurement used in the analysis. LDI scores are scaled such that higher values indicate lower likelihood of dementia.

#### Covariates

*Education* was based on the International Standard Classification of Education (ISCED; UNESCO, 2012). For all individuals, a score of 1 was given for those who completed primary education, a score of 2 was given for those who completed up to a lower secondary education (grades 7–9), a score of 3 was given to those who completed an upper secondary education (grades 10–12 or GED), a score of 4 was given to those who completed post-secondary non-tertiary education or short-cycle tertiary education (e.g., vocational school, associate’s degree), a score of 5 was given to those who completed a bachelor’s degree (or equivalent), and a score of 6 was given to those who completed a master’s degree or higher.

*Depressive symptomatology* was measured using the Center for Epidemiologic Studies – Depression (CESD) scale (Radloff 1977) in SATSA and the Cambridge Mental Disorders of the Elderly Examination (CAMDEX; Roth et al. 1986) in LSADT which was modified to include additional items capturing depression history and current affective state (McGue and Christensen 1997). The CES-D contains 20 items each scored between 0 and 3. The numeric value represents the frequency of experiencing the given depression symptom listed for that item during the past week. The scale contains four items that were reverse-scored such that higher values indicate more severe depression symptoms. The modified CAMDEX contains 19 items that were scored on a Likert scale between 1 and 3 and two dichotomous items. IRT methods were applied to the CES-D scores to harmonize scores for depressive symptomatology (Gatz et al. 2015). The harmonized CES-D scale scores, thus, range from 17 to 49 with 17 representing a score of 0 and 49 representing the maximum score.

*Follow-up time* between subjective ratings of health and the cognitive and functional ability assessments that were used to estimate LDI scores were also included in the present analyses. These scores were calculated by subtracting the reported age of the twins at their subjective health measurement occasion from their reported age at the in-person assessment from which LDI scores were computed. Follow-up time is reported in years.

### Data analysis

Analysis of SATSA and LSADT data included six steps. First, we computed descriptive statistics for each sample, including means, standard deviations, and correlations for subjective health and LDI variables. Phenotypic correlations present the magnitude and direction of the association between each subjective health measure and LDI scores.

Second, we tested whether subjective health predicted LDI scores. Phenotypic regressions were estimated for each subjective health variable in both datasets: SRH, COMP, and ACT in SATSA and SRH in LSADT. Standard errors were corrected for dependence of twins in the same family. We compared the model fits of our baseline phenotypic regressions to models that included age, sex, education, depressive symptomatology, and follow-up time.

Third, twin correlations were presented to show the presence and absence of additive genetic (A), shared-environmental (C) and nonshared environmental (E) influences underlying each subjective health variable, the LDI, and the covariance between them. Additive genetic variance constitutes the cumulative effect of genotype that makes two twins from the same family similar on a phenotype (e.g., subjective health). Shared (or common) environmental variance refers to any non-genetic source of variance that makes twins from the same family similar on a phenotype. Finally, nonshared environmental variance is any factor that makes two (identical) twins dissimilar to each other on a phenotype. The presence of the ACE components is determined by comparing MZ twin correlations to DZ twin correlations. If MZ twin-correlations are greater than DZ twin-correlations, genetic variance accounts for variance in subjective health or LDI. Similarly, if the MZ twin-correlations are not at least twice as great as the DZ twin correlations, shared-environmental effects are present. MZ twin-correlations less than 1 show that non-shared environmental variance accounts for differences in subjective health, LDI, or their association. In addition, cross-twin cross-trait correlations between each subjective health variable and LDI were estimated and are interpreted the same as univariate twin correlations.

**Fig. 1:**
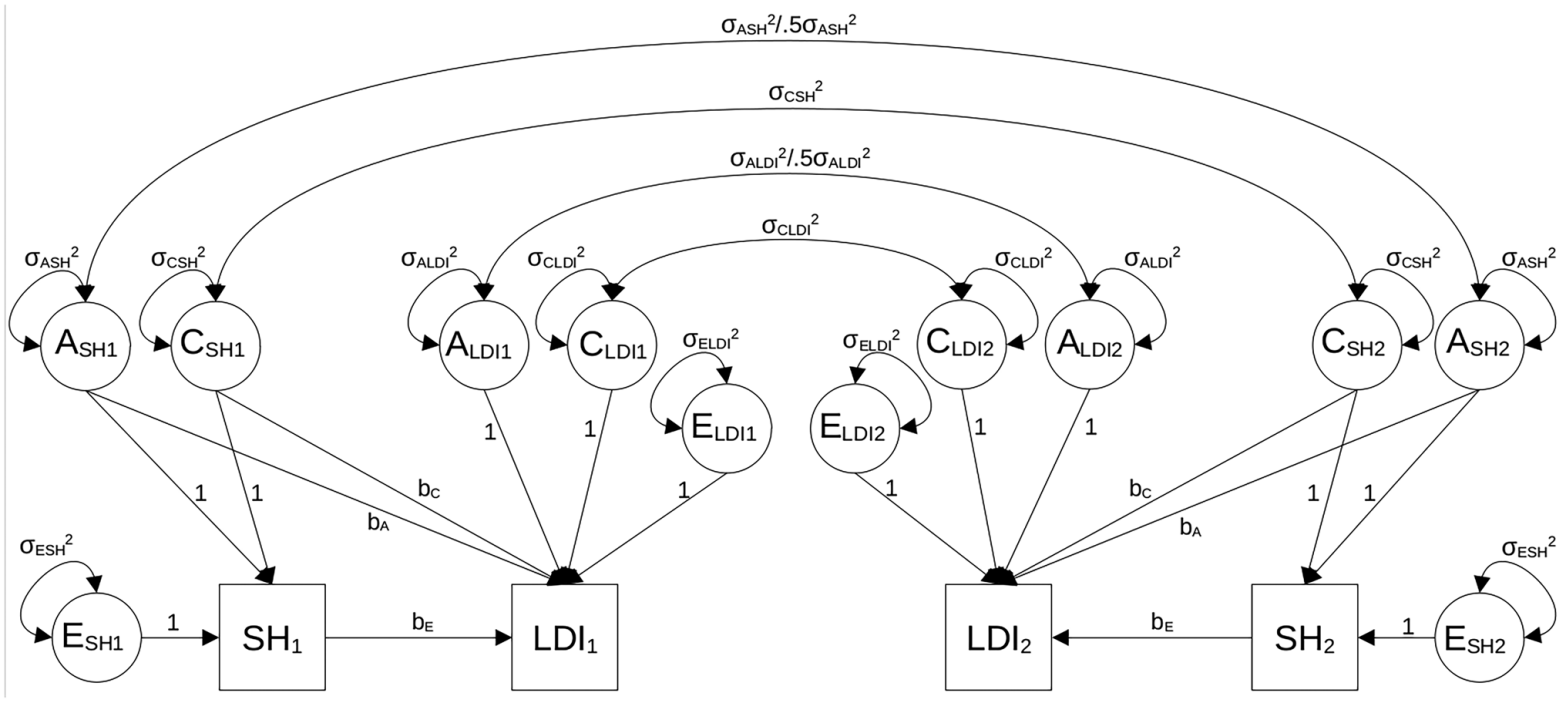
Bivariate ACE model for the association between subjective health and latent dementia index. *Note.* SH = subjective health rating measure; LDI = latent dementia index; A = additive genetic variance; C = shared environmental variance; E = nonshared environmental variance; subscripts 1 and 2 refer to twins 1 and 2. The MZ covariance is equal to the variance, σ^2^_A_, so that correlation between A_1_ and A_2_ is equal to 1.0. The DZ covariance is 0.5σ^2^_A_ so that the correlation between A_1_ and A_2_ is equal to 0.5

Fourth, we fit classical univariate ACE models to estimate the proportion of variance for each phenotype attributed to genetic, shared environmental, and nonshared environmental variance. Classical twin modeling assumptions were met by fixing the covariances between twin 1’s ACE components and twin 2’s ACE components appropriately (Fig. 1). To satisfy the assumption that MZ twins share their entire genotype whereas DZ twins share 50% of their segregating genes, on average, the covariance between A1 and A2 of subjective health was fixed to σ_A_^2^ in the MZ group and 0.5*σ_A_^2^ in the DZ group. The covariance between C1 and C2 of subjective health was fixed to σ_C_^2^ in both the MZ and DZ groups to reflect the assumption that shared environments influence twins equally regardless of zygosity. Nonshared environmental variance (E) was estimated as the residual variance of the subjective health measure and is uncorrelated between twins, an approach equivalent to “standard” SEM-based ACE variance decompositions (Loehlin 1996).

We also explored the possibility of an ADE model. In this model, a dominant genetic variance component (D) replaces the shared environmental variance component and assumes that dizygotic twins' dominant genetic components are correlated 0.25. In the case of models including genetic dominance (ADE), we fixed the D-covariance to σ_D_^2^ in the MZ group and 0.25*σ_D_^2^ in the DZ group. In addition to the three assumptions described above, classical ACE models also assume that ACE (or ADE) components are uncorrelated, do not interact, and non-assortative mating of twins’ parents. We fixed the A, C, D, and E factor loadings on the subjective health variable to 1 to estimate their variance.

Fifth, we fit bivariate ACE models to subjective health and LDI (Fig. 1). The variance of each subjective health variable was decomposed into ACE (or ADE) variance components as described above. LDI was then regressed onto the A and C variance components of subjective health (b_A_ and b_C_) as well as the residual (nonshared environmental) variance in SH_1_ and SH_2_ unaccounted by A and C. In this way, b_E_ represents the association between subjective health and LDI after statistically adjusting for genetic and shared environmental confounds. Residual variances of the LDI variables were estimated as A, C, and E latent variance components in a manner identical to subjective health.

The genetic and environmental correlations between subjective health and LDI were estimated where possible as additional parameters as a function of the genetic and environmental variance components. We used the following expressions, shown for additive genetic (*r*_*G*_) and nonshared environmental (*r*_*E*_) components;

1 $$r_G = b_A \sigma_A^2 /\sqrt {{\left( {\sigma_A^2 *\left( {b_A^2 \sigma_A^2 + \sigma_{ALDI}^2 } \right)} \right)}}$$

2 $$r_E = b_E \sigma_E^2 /\sqrt {{\left( {\sigma_E^2 *\left( {b_E^2 \sigma_E^2 + \sigma_{ELDI}^2 } \right)} \right)}}$$

These correlations provide effect sizes of the additive genetic factors and environmental factors that overlap respectively between subjective health measures and LDI. We also present bivariate heritability and environmental estimates, which are the proportion of the phenotypic association between subjective health and dementia risk that is due to overlapping genetic and environmental factors, respectively.

We followed a stepwise model fitting sequence, starting with the full model (fewest degrees of freedom) and removing selected parameters until we achieved the most parsimonious model without significant loss of model fit. The baseline model for all three subjective health variables was an ACE model in which the variance of subjective health was decomposed into ACE variance components onto which twins’ LDI scores were regressed. Baseline models included the effect of age on both LDI and subjective health to minimize the confounding effects of age on the A and C covariances. Subsequent models tested whether the regression effects of the ACE components underlying subjective health on LDI (b_A_, b_C_, and b_E_ in Fig. 1) could be set to zero. We also tested whether the genetic and shared environmental confounds could be equated or fixed to zero. The test of greatest interest in the current study was whether the E effects underlying subjective health on LDI were statistically significantly different from zero. Rejection of the null hypothesis provides support for the quasi-causal effect of subjective health on LDI. A final model was tested in which we added the covariates to the best fitting model after initial model selection.

Finally, to account for sex differences in dementia risk, the sixth step in our analysis tested a series of sex-limitation models. The sex-limitation models allowed all variance components and regressions to be estimated freely across sex of twins. These models included only four groups—MZ males, MZ females, DZ males, and DZ females—because SATSA did not collect opposite-sex DZ pairs. We then tested whether a model in which the ACE variances and ACE regression effects were fixed to be the same across sex was statistically equivalent to the baseline model. In all analyses, we used an alpha cut-off value of .05. Both unadjusted and adjusted parameter estimates are reported.

Models were compared using Satorra-Bentler corrected χ^2^ tests for nested models (Satorra et al. 2001), the Akiake Information Criterion (AIC), and the Bayesian Information Criterion (BIC; Burnham and Anderson 2004). All models were estimated in M*plus* 8.2 (Muthen and Muthen 2017) using maximum likelihood with robust standard errors.

## Results

### Phenotypic effects of subjective health on dementia risk

Sample statistics are presented in Table 1. The mean scores in SATSA for SRH, COMP, ACT were near the *T*-score mean of 50. Mean LDI for SATSA, higher scores of which indicate lower dementia risk, was 7.30, which suggests that likelihood of dementia tended to be low across the sample given that the cutoff score established in prior validation studies for dementia is 5.26 (Beam et al. 2022). In LSADT, the mean of SRH was 48.82 (*SD* = 9.41) and did not statistically differ from SATSA. The mean of LDI in LSADT was 6.14 and was statistically different from SATSA. We note that the mean value was above the cutoff score for probable diagnosis of dementia. The average time in years between subjective health measurement and LDI measurement in SATSA was 22.83 (*SD* = 4.35) whereas in LSADT it was 3.59 (*SD* = 2.70). SATSA and LSADT samples differed in age, LDI scores, depressive symptomatology, and education; however, aside from age, raw differences were small.

**Table 1:** Descriptive statistics

Variable	SATSA			LSADT			p
	*N*	Mean (*SD*)	Range	*N*	Mean (*SD*)	Range	
Age	548	54.95 (10.01)	35.90–78.90	4,373	77.32 (5.34)	70.00–102.00	< .001
% Female	548	60	–	4,373	59	–	.500
LDI	548	7.30 (1.51)	1.12–10.90	4,373	6.14 (0.64)	4.23–8.28	< .001
SRH	548	48.39 (8.91)	42.24–78.06	3,115	48.82 (9.41)	38.49–78.96	.300
COMP	544	49.83 (8.95)	35.30–70.50	–	–	–	–
ACT	547	47.74 (8.19)	43.05–73.42	–	–	–	–
Depressive Symptoms	545	23.10 (4.72)	17.00–42.10	4,373	21.23 (4.77)	17.00–47.00	< .001
Education	548	2.07 (1.55)	1.00–6.00	4,373	2.81 (1.68)	1.00–9.00	< .001
Follow-Up Time (Years)	548	22.83 (4.35)	9.80–27.33	3,115	3.59 (2.70)	1.00–10.00	< .001

*Note*. LDI = latent dementia index, SRH = Self-Rated Health, COMP = Comparative Health, ACT = Health Impact on Activities, Follow-Up Time = the number of years between self-rated health assessments and cognitive and functional ability assessments used to estimate LDI scores in each sample, *N* = number of unique observations for a given variable

Phenotypic correlations in SATSA between LDI and SRH, COMP, and ACT were − .08, − .02, and − .10 respectively and in LSADT the correlation between SRH and LDI was − .26 (Table 3).

Phenotypic regression models in both datasets were compared with models that included covariates. Across both data sets and all subjective health variables, the inclusion of covariates significantly improved model fit. The best fitting models included age, sex, education, depressive symptomatology, and follow-up time for SRH (χ^2^_5_ = 116.92, *p* < .001), COMP (χ^2^_5_ = 123.83, *p* < .001), and ACT (χ^2^_5_ = 115.88, *p* < .001) in SATSA as well as SRH (χ^2^_5_ = 578.73, *p* < .001) in LSADT.

Results of the phenotypic models are reported in Table 2. In SATSA, SRH (*b* = − 0.02, *p* < .05) and ACT (*b* = − 0.03, *p* < .01) but not COMP (*b* = − 0.01, *p* = .33) were negatively associated with LDI. After controlling for age, sex, education, depressive symptomatology, and follow-up time, none of these associations remained statistically significant (SRH: *b* = 0.002, *p* = .829; COMP: *b* = 0.001, *p* = .865; ACT: *b* = − 0.01, *p* = .409). In LSADT, SRH negatively predicted LDI (*b* = − 0.02, *p* < .001) even once covariates were adjusted for (*b* = − 0.01, *p* < .001). Given the lack of association between COMP and LDI at the phenotypic level, we did not proceed with further analyses with the COMP variable.

**Table 2:** Unstandardized estimates from regression models predicting LDI from subjective health measures

Parameter	SATSA						LSADT	
	SRH	SRH Adj	COMP	COMP Adj	ACT	ACT Adj	SRH	SRH Adj
	Est./ 95% CI	Est./ 95% CI	Est./ 95% CI	Est./ 95% CI	Est./ 95% CI	Est./ 95% CI	Est./ 95% CI	Est./ 95% CI
b_SH_	− 0.02* / [− 0.03, − 0.00]	0.002/ [− 0.01, 0.02]	− 0.01 / [− 0.02, 0.01]	0.001 / [− 0.01, 0.01]	− 0.03** / [− 0.04, − 0.01]	− 0.01 / [− 0.02, 0.01]	− 0.02*** / [− 0.02, − 0.02]	− 0.01*** / [− 0.01, − 0.01]
b_Age_	−	0.07 / [− 0.08, 0.21]	−	0.07 / [− 0.08, 0.22]	–	0.07 [− 0.07, 0.21]	–	− 0.12*** / [− 0.15, − 0.09]
b_Sex_	–	0.33**/ [0.09, 0.57]	–	0.33** / [0.09, 0.57]	–	0.33** / [0.09, 0.57]	–	0.15***/ [0.11, 0.18]
b_Education_	–	0.14*** / [0.07, 0.21]	–	0.14*** / [0.07, 0.21]	–	0.14*** / [0.07, 0.21]	–	0.08***/ [0.07, 0.09]
b_Dep_	–	− 0.03* / [− 0.06, − 0.01]	–	− 0.03* / [− 0.06, − 0.01]	–	− 0.03* / [− 0.05, − 0.004]	–	− 0.03*** / [− 0.03, − 0.02]
b_Follow_	–	0.14*** / [0.11, 0.17]	–	0.14*** / [0.11, 0.17]	–	0.14*** / [0.11, 0.17]	–	0.04*** / [0.04, 0.05]
LDI residual	2.26*** / [1.97, 2.55]	1.80*** / [1.55, 2.05]	2.28*** / [1.99, 2.57]	1.80*** / [1.55, 2.05]	2.25*** / [1.96, 2.54]	1.80***/ [1.55, 2.05]	0.39*** / [0.37, 0.41]	0.34*** / [0.32, 0.35]

*Note*. SRH = Self-Rated Health, COMP = Comparative Health, ACT = Health Impact on Activities, Adj = Adjusted, *b*_*SH*_ = effect of subjective health measures, *b*_*Age*_ = effect of age, *b*_*Sex*_ = effect of sex, *b*_*Education*_ = effect of education, *b*_*Dep*_ = effect of depressive symptomatology, *b*_*Follow*_ = effect of follow-up time, LDI = Latent Dementia Index

^*^ p < .05, ** p < .01, *** p < .001

### Multivariate twin analyses for subjective health and dementia risk

Table 3 presents the univariate and cross-twin cross-trait correlations between subjective health and LDI. MZ twin correlations for SRH and LDI were larger than DZ twin correlations in both SATSA and LSADT, suggesting the presence of additive genetic effects on the SRH-LDI relationship. The same was true with ACT in SATSA. Cross-twin cross-trait correlations show genetic confounding between LDI and subjective health variables in SATSA and LSADT.

**Table 3:** Cross-twin cross-trait twin correlation estimates for LDI and subjective health variables in SATSA and LSADT

	SATSA				LSADT			
	LDI T1	SRH T1	LDI T2	SRH T2	LDI T1	SRH T1	LDI T2	SRH T2
LDI Twin 1	1	*−* *.08*	.23*	*−* *.08*	1	*−* .26***	.29***	*−* *.04*
SRH Twin 1	− 0.08	1	*−* *.08*	*.12*	− .26***	1	*−* *.04*	*.04*
LDI Twin 2	.48 ***	− .05	1	*−* *.08*	.46***	− .17***	1	*−* .26***
SRH Twin 2	− .05	.37**	− .08	1	− .17***	.26***	− .26***	1

	LDI Twin 1	ACT T1	LDI T2	ACT T2				
LDI T1	1	*−* .10*	.23*	*−* *.05*				
ACT T1	− .10*	1	*−* *.05*	.30***				
LDI T2	.48***	− .08	1	*−* .10*				
ACT T2	− .08	.44***	− .10*	1				

*Note.* LDI = latent dementia index, SRH = Self-Rated Health, ACT = Health Impact on Activities, T = Twin. Monozygotic (MZ) twin estimates are shown in non-italic value below the diagonal, dizygotic (DZ) twin estimates are shown in italic value above the diagonal

^*^ p < .05, ** p < .01, *** p < .001

Next, we fit the univariate ACE models to the SRH and LDI variables. Model fitting results and parameter estimates are presented in Supplementary Table 1. The best fitting univariate models in SATSA for SRH, COMP, ACT and LDI were AE (χ^2^_1_ = 0.510, *p* = .475), CE (χ^2^_1_ = 0.435, *p* = .510), A = C (χ^2^_1_ = 0.132, *p* = .717), and AE (χ^2^_1_ = 0.000, *p* = .999) models respectively. In LSADT, the best fitting model for SRH was an AE model (χ^2^_1_ = 3.010, *p* = .083) whereas for LDI, a model in which A variance could not be distinguished from C variance (A = C model) fit the data best (χ^2^_1_ = 1.015, *p* = .308). In all cases, we did not find evidence for dominant genetic influence on any phenotype.

Model fitting results for bivariate ACE models are presented in Table 4. In SATSA, across all subjective health variables, the C components of LDI could be dropped. As such, the best fitting model for SRH was a more parsimonious AE model in which only genetic confounding between SRH and LDI was present (Model 6: χ^2^_4_ = 1.050, *p* = .902). For ACT, a more parsimonious model in which A and C variances were set equal and the quasi-causal effect of ACT on LDI was dropped emerged as the best fitting model (Model 6: χ^2^_4_ = 0.554, *p* = .968). In all SATSA models, adjustment for age, sex, education, depressive symptomatology, and follow-up time improved model fit. For LSADT, the best fitting model did not include C variance for SRH (AE), equated A and C variance for LDI, and included both additive genetic and nonshared environmental effects of SRH on LDI (Model 5: χ^2^_3_ = 4.054, *p* = .256). As with SATSA, adjustment with covariates improved model fit.

**Table 4:** Model fit results for bivariate effects of ACE parameters of subjective health on LDI

Model	*χ*^2^	Δ*df*	*p*	AIC	BIC
SATSA					
SRH					
1. ACE_SRH_, ACE_LDI_	–	–	–	16,696.99	16,784.99
2. ACE_SRH_, AE_LDI_	0.060	1	.806	16,695.08	16,779.25
3. AE_SRH_, AE_LDI_	1.037	3	.792	16,692.17	16,768.69
4. A = C_SRH_, AE_LDI_	2.050	3	.562	16,693.33	16,769.85
5. CE_SRH_, AE_LDI_	3.191	3	.363	16,694.63	16,771.15
6. *AE*_*SRH*_*, AE*_*LDI*_*, no b*_*E*_	*1.050*	*4*	*.902*	*16,690.17*	*16,762.86*
7. Adjusted*	38.005	4	< .001	16,659.83	16,747.83
ACT					
1. ACE_ACT_, ACE_LDI_	–	–	–	16,577.26	16,665.26
2. ACE_ACT_, AE_LDI_	0.000	1	.999	16,575.26	16,659.44
3. AE_ACT_, AE_LDI_	0.469	3	.926	16,571.76	16,648.28
4. A = C_ACT_, AE_LDI_	0.169	3	.982	16,571.46	16,647.98
5. CE_ACT_, AE_LDI_	1.335	3	.721	16,572.85	16,649.37
6. *A* = *C*_*ACT*_*, AE*_*LDI*_*, no b*_*E*_	*0.554*	*4*	*.968*	*16,569.90*	*16,642.60*
7. Adjusted*	36.944	4	< .001	16,540.28	16,628.28
LSADT					
SRH					
1. ACE_SRH_, ACE_LDI_	–	–	–	101,082.61	101,222.834
2. ACE_SRH_, AE_LDI_	2.427	1	.119	101,084.15	101,218.28
3. ACE_SRH_, CE_LDI_	3.526	1	.060	101,082.29	101,216.42
4. ACE_SRH_, A = C_LDI_	0.000	1	.999	101,080.61	101,214.74
5.* AE*_*SRH*_*, A* = *C*_*LDI*_	*4.054*	*3*	*.256*	*101,080.72*	*101,202.66*
6. A = C_SRH_, A = C_LDI_	8.689	3	.034	101,085.77	101,207.71
7. CE_SRH_, A = C_LDI_	12.359	3	.006	101,090.00	101,211.94
8. AE_SRH_, A = C_LDI_, no b_E_	18.430	4	.001	101,092.97	101,208.81
9. Adjusted*	376.169	4	< .001	100,687.69	100,834.01

*Note.* Best fitting models without adjusting for covariates are shown in italics. ACE parametrization in Model column listed for Subjective Health then Latent Dementia index. SRH = Self-Rated Health; ACT = Health Impact on Activities; Adjusted = Adjusted for covariates age, sex, education, depressive symptomatology, and follow-up time; Δ*df* = difference in degrees of freedom between compared models; *p* = probability value; AIC = Akaike Information Criterion; BIC = Bayesian Information Criterion; A = additive genetic variance; E = nonshared environmental variance; *b*_*E*_ = nonshared environmental effect

^*^Comparison model for adjusted models is the best fitting unadjusted model

Unstandardized bivariate ACE parameter estimates for SATSA and LSADT are reported in Table 5 (Standardized estimates are reported in Supplementary Table 2). In SATSA, SRH did not significantly predict LDI, when not adjusting for covariates (*b*_*A*_ = − 0.04, *p* = .148) or after their inclusion (*b*_*A*_ = − 0.01, *p* = .532). The A component of ACT, however, was negatively associated with LDI (*b*_*A*_ = − 0.04, *p* < .05) but this effect was no longer significant after controlling for covariate effects (*b*_*A*_ = − 0.03, *p* = .177). Of the covariates, sex (*b*_*sex*_ = 0.31, *p* < .05), education (*b*_*edu*_ = 0.15, *p* < .001), depressive symptomatology (*b*_*dep*_ = − 0.03, *p* < .05), and follow-up time (*b*_*follow*_ = 0.07, *p* < .001) significantly predicted LDI in adjusted models whereas age did not (*b*_*age*_ = − 0.11, *p* = .143).

**Table 5:** Bivariate effects of ACE parameter estimates of subjective health on LDI

Param	SATSA				LSADT	
	SRH	SRH Adj	ACT^†^	ACT Adj.^†^	SRH	SRH Adj
b_A_	− .04 / [− .09, .01]	− .01/ [− .06, .03]	− .04* / [− .08, − .003]	− .03 / [− .06, .01]	− .05*** / [− .07, − .02]	− .02* / [− .05, − .003]
b_E_	–	–	–	–	− .01*** / [− .01, − .005]	− .01* / [− .01, .000]
b_Age_	− .30*** / [− .43, − .17]	− .11 / [− .26, .04]	− .30*** / [− .43, − .17]	− .11/ [− .26, .04]	− .17*** / [− .20, − .13]	− .12*** / [− .15, − .08]
b_Sex_	–	0.31* / [.06, .56]	–	.31* / [.06, .56]	–	.15*** / [.11, .19]
b_Education_	–	.15*** / [.08, .23]	–	.15*** / [.08, .22]	–	.07*** / [.06, .08]
b_Dep_	–	− .03* / [− .05, − .001]	–	− .03* / [− .05, − .001]	–	− .03*** / [− .03, − .02]
b_Follow_	–	.07*** / [.03, .12]	–	.07*** / [.03, .11]	–	0.03*** / [.02, .04]

*Note.* Estimates reported for best fitting models with and without adjustment for covariates. Covariates include age, sex, education, and depression. Age is included in all baseline models. SRH = Self-Rated Health; ACT = Health Impact on Activities; Adj = Adjusted, *b*_*A*_ = genetic effect of subjective health, *b*_*E*_ = nonshared environment effect of subjective health, *b*_*Age*_ = effect of age, *b*_*Sex*_ = effect of sex, *b*_*Education*_ = effect of education, *b*_*Dep*_ = effect of depressive symptomatology, *b*_*Follow*_ = effect of follow-up time

^†^A and C subjective health variances are indistinguishable in these models. *b*_*A*_ = represents effect of familial confounding

^*^ *p* < .05, ** *p* < .01, *** *p* < .001

In LSADT, the E component of SRH predicted LDI (*b*_*E*_ = − 0.01, *p* < .001) and remained statistically significant after adjusting for covariates (*b*_*E*_ = − 0.01, *p* < .05). The A component of SRH also negatively predicted LDI (*b*_*A*_ = − 0.05, *p* < .001), and this association remained significant after covariates were included (*b*_*A*_ = − 0.02, *p* < .05). Age (*b*_*age*_ = − 0.12, *p* < 0.001), sex (*b*_*sex*_ = 0.15, *p* < .001), education (*b*_*edu*_ = 0.07, *p* < .001), depressive symptomatology (*b*_*dep*_ = − 0.03, *p* < .001), and follow-up time (*b*_*follow*_ = 0.3, *p* < .001) were predictive of LDI in LSADT.

In SATSA, fully adjusted models revealed a small, non-significant genetic correlation between LDI and SRH (*r*_*G*_ = − .09, 95% *CI* [− .36, .18]). Genetic correlations were not computed for ACT given the lack of identifiable additive genetic variance. Since best-fitting models in SATSA did not contain a nonshared environmental effect (an E regression), environmental correlations were 0. Bivariate heritability, thus, was 100%. In LSADT, small nonshared environmental (*r*_*E*_ = − .09, 95% *CI* [− .17, − .01]) and genetic (*r*_*G*_ = − .39, 95% *CI* [− .69, − .02]) correlations were observed in the fully adjusted model. The proportion of the phenotypic correlation attributable to nonshared environmental factors is 0.46 (*p* < .05) whereas the proportion attributable to genetic confounding is 0.54 (*p* < .05).

Sex-limitation analysis did not reveal evidence for any differences in effects of interest between male and female twin pairs (Supplementary Table 3). In SATSA, all parameters could be equated across sex groups without loss of model fit (SRH: χ^2^_11_ = 12.9, *p* = .300; ACT: χ^2^_11_ = 12.25, *p* = .345). In LSADT, all regression parameters (A, C, and E effects) could be equated across sex without loss of model fit (SRH: χ^2^_3_ = 5.73, *p* = .126) suggesting that the effect of subjective health on LDI does not differ between men and women.

## Discussion

The present study investigated whether subjective health, measured with three different items, predicted latent dementia risk and whether genetic variance, environmental variance, particularly the nonshared environment, or both accounted for their association. We found that subjective health measured with a global item was negatively associated with dementia risk even after adjusting for age, sex, education, depressive symptomatology, and follow-up time and that this association was primarily accounted for by common genetic variance. Yet, although the observed genetic correlation was larger than the nonshared environmental correlation, nonshared environmental pathways still contributed significantly to the association. We also found that subjective health, measured relative to how much it impairs activities, predicted latent dementia risk but that this association does not remain after adjusting for observed covariates. Finally, comparative health ratings were not associated with dementia risk. Results for comparative ratings of health typically differ from global health ratings and ratings of health impact on activities (Franz et al. 2017; Finkel et al. 2020), reflecting the different frame of reference by virtue of directly comparing individuals to their same-aged peers.

Individuals’ awareness of their physical health may predict their likelihood of dementia. Previous studies have found that poorer self-rated health increases risk for dementia (Ganguli et al. 2019; Hamid et al. 2010; Jia et al. 2020; John and Montgomery 2013; Lipnicki et al. 2019; Montlahuc et al. 2011; Sargent-Cox et al. 2011; Stephan et al. 2021; Weisen et al. 1999; Yip et al. 2006) but have been limited to controlling for common measured confounds only. In this way, our study extends prior work by estimating the degree to which subjective health predicts dementia risk after adjusting for the genetic confounds underlying their association. Our results suggest that the association is partially confounded by additive genetic effects shared by subjective health and dementia. Nevertheless, a significant portion of the phenotypic association is attributable to nonshared environmental factors, consistent with one previous study which found that those who rate their health as poorer have increased risk for developing dementia, even after adjusting for Alzheimer's disease polygenic scores (Stephan et al. 2021). Although polygenic scores account for the total variance in a trait explained by the summed effects of individual genes, they do not adjust for unmeasured genetic confounds or provide an estimate of how much the association is due to shared heritability. In this way, our study provides a more robust demonstration that self-rated health and dementia risk are significantly associated despite the presence of genetic confounding.

As in prior studies, a moderate amount of the variance in subjective health was attributed to additive genetic variance (Franz et al. 2017; Harris et al. 2017; Mosing et al. 2010; Silventoinen et al. 2007; Svedberg et al. 2001). In LSADT, the genetic component accounted for part of the total variance in dementia risk, suggesting that the same genetically influenced characteristics that account for better or worse health may also explain differences in dementia risk. We note, however, that the current findings do not explain which genetic mechanisms or genetically-influenced mechanisms account for the association between subjective health measures and dementia risk.

Also in LSADT, we observed a significant association between self-rated health and dementia risk, indicating that at least some of the variance in dementia risk is accounted for by environmental variance underlying subjective health. One possibility is that self-rated health measures capture within-family variance in environmentally-driven health processes (e.g., cardiovascular disease, obesity, and diabetes; see Albanese et al. 2017; Eriksson et al. 2010; Xue et al. 2019) that in turn account for variance in dementia and dementia risk. A second possibility is that within-pair differences in self-rated health may be a proxy for pair differences in personality-based appraisals of health (e.g., more neurotic twins may appraise their health as worse than their less neurotic co-twins) that account for differences in dementia risk. However, the nonshared environmental correlation was small, as the effect was diminished after adjusting for covariates. Although outside of the purpose of the current study, there may be an interaction between subjective health and one or more of these covariates. For example, in the case of educational attainment, individuals with higher education tend to be wealthier and can afford to make decisions that prolong good health (Ross and Chia-Ling, 1995).

Genetic confounding of the utility of self-reported patient measures in predicting dementia may not be unique to subjective health. Subjective memory concerns have been proposed as a symptom of cognitive decline and dementia (Jessen et al. 2014). However, a recent twin study showed that they are characterized by a heritable, trait-like component and are more strongly related to objective memory ability through genetic rather than environmental pathways (Bell et al. 2023). Our results reflect a similar phenomenon whereby self-reported information conveyed by individuals may have substantial genetic influence that confounds its association with dementia outcomes.

Inclusion of age, sex, education, depressive symptomatology, and follow-up time rendered additive genetic effects of subjective health on dementia risk null in one sample and reduced their magnitude substantially in another. One possible cause is an unmodeled interaction between the additive genetic component of subjective health and one or more of the covariates. For example, depressive symptomatology is known to be associated with subjective health (Ambresin et al. 2014; Mulsant et al. 1997) and the nature of depressive symptoms tend to become more somatic as individuals age (Fiske et al. 2009). It may be that unmodeled genetic overlap between depressive symptomatology and subjective health accounts for some portion of variance in dementia risk which in our results manifested as attenuation of the main effects. Similar explanations may be plausible for diminished effects of subjective health on LDI due to the addition of sex, which is also known to predict subjective health (Idler 2003).

Although we found a similar direction of effects of subjective health on dementia risk in SATSA and LSADT, associations between subjective health and LDI differed in their significance between our two datasets. One possible explanation is that the analytical sample size in SATSA was much smaller than in LSADT, which possibly increased Type II error rates and lowered power. This is suggested by the fact that estimates in SATSA were of similar magnitude and direction to those from LSADT but were not statistically significant. Alternatively, the number of years between subjective health measurements and dementia risk measurements in SATSA was much greater than in LSADT. As the measurement interval could have been over 20 years in SATSA whereas only 8–10 years in LSADT, the passage of time may have rendered the phenotypic correlation between subjective health and dementia risk too small to detect statistically significant effects. Indeed, follow-up time was predictive of LDI in both samples, but was more highly correlated in SATSA than LSADT. Furthermore, the addition of covariates, which included follow-up time, accounted for the entire bivariate association between subjective health and LDI in SATSA but not in LSADT. A final possibility is that the lower age of the SATSA sample similarly reduced the size of the correlation.

The results of our study must be considered within the context of its limitations. First, quantitative genetic models assume that environmental similarity does not differ across zygosity types, non-assortative mating of parentage, and independent genetic and environmental variance components. The degree to which these assumptions are false in the data can lead to bias in estimates and interpretation. Second, our study used dementia risk operationalized by a latent risk factor estimated from cognitive and functional ability data that has been separately validated (Beam et al. 2022). We used this outcome due to limited diagnostic availability in the selected data samples, however, this decision somewhat limits direct comparability to studies using clinical gold standard dementia diagnoses.

In summary, our study provides further support for the association between subjective health and dementia risk while also providing evidence that this association is explained by both genetic and environmental factors shared between the two traits. Although not as a large as the genetic effect, the significant nonshared environmental correlation between self-rated health and likelihood of dementia supports the hypothesis that worsening health may differentially predict likelihood of an eventual dementia diagnosis. Further studies should assess whether the main health features captured by subjective health items are similarly correlated at the genetic level with dementia outcomes as this will likely lead to a better understanding of which health risk factors of dementia are in fact modifiable. Moreover, given the increasing international burden that dementia poses, future studies should also consider whether individuals’ perceptions of their declining health can be used to make accurate predictions about whether they will be diagnosed with dementia.

### Supplementary Information

Below is the link to the electronic supplementary material.

Supplementary file1 (DOCX 12 KB)
