# Supplementary material for: Prospective Effects of Self-Rated Health on Dementia Risk in Two Twin Studies of Aging
Source: Behav Genet. 2024 Jun 1;54(4):307–20. doi: 10.1007/s10519-024-10182-1 (PMC11196327; doi:10.1007/s10519-024-10182-1)
Supplement: Supplementary file 1 — Supplementary file1 (DOCX 12 KB) [file 10519_2024_10182_MOESM1_ESM.docx]

**Supplementary Table 1**

*Standardized Parameter Estimates and Goodness of Fit Statistics for Univariate Twin Models*

| Model | a^2^ | c^2^ | e^2^ | d^2^ | *χ*^2^ | Δ*df* | *p* | AIC | BIC |
| --- | --- | --- | --- | --- | --- | --- | --- | --- | --- |
| **SATSA** |  |  |  |  |  |  |  |  |  |
| SRH |  |  |  |  |  |  |  |  |  |
| ACE | 0.53 | -0.15 | 0.62 | - | - | - | - | 3,935.67 | 3,954.80 |
| ADE | 0.08 | - | 0.62 | 0.30 | - | - | - | 3,935.67 | 3,954.80 |
| *AE* | *0.34* | *-* | *0.66* | *-* | *0.510* | *1* | *.475* | *3,934.25* | *3,949.55* |
| CE | - | 0.21 | 0.79 | - | 3.073 | 1 | .080 | 3,937.48 | 3,952.78 |
| A=C | 0.14 | 0.14 | 0.73 | - | 1.825 | 1 | .177 | 3,935.89 | 3,951.19 |
| COMP |  |  |  |  |  |  |  |  |  |
| ACE | -0.22 | 0.27 | 0.95 | - | - | - | - | 3,927.29 | 3,946.42 |
| ADE | 0.60 | - | -0.55 | 0.95 | - | - | - | 3,927.29 | 3,946.42 |
| AE | 0.14 | - | 0.86 | - | 1.585 | 1 | .208 | 3,926.93 | 3,942.24 |
| *CE* | *-* | *0.13* | *0.87* | *-* | *0.435* | *1* | *.510* | *3,925.82* | *3,941.12* |
| A=C | 0.07 | 0.07 | 0.86 | - | 0.828 | 1 | .363 | 3,926.23 | 3,941.54 |
| ACT |  |  |  |  |  |  |  |  |  |
| ACE | 0.30 | 0.13 | 0.57 | - | - | - | - | 3,818.07 | 3,837.20 |
| ADE | 0.69 | - | 0.57 | -0.26 | - | - | - | 3,818.07 | 3,837.20 |
| AE | 0.46 | - | 0.54 | - | 0.492 | 1 | .483 | 3,816.59 | 3,831.89 |
| CE | - | 0.33 | 0.67 | - | 1.089 | 1 | *.297* | 3,817.59 | 3,832.89 |
| *A=C* | *0.20* | *0.20* | *0.60* | *-* | *0.132* | *1* | *.717* | *3,816.24* | *3,831.54* |
| LDI |  |  |  |  |  |  |  |  |  |
| ACE | 0.47 | 0.00 | 0.53 | - | - | - | - | 1,974.37 | 1,993.50 |
| ADE | 0.47 | - | -0.00 | 0.53 | - | - | - | 1,974.37 | 1,993.50 |
| *AE* | *0.47* | *-* | *0.53* | *-* | *0.000* | *1* | *.999* | *1,972.37* | *1,987.67* |
| CE | - | 0.34 | 0.67 | - | 3.320 | 1 | .068 | 1,976.03 | 1,991.34 |
| A=C | 0.21 | 0.21 | 0.59 | - | 1.064 | 1 | .302 | 1,973.57 | 1,988.87 |
| **LSADT** |  |  |  |  |  |  |  |  |  |
| SRH |  |  |  |  |  |  |  |  |  |
| ACE | 0.46 | -0.21 | 0.75 | - | - | - | - | 22,779.32 | 22,808.32 |
| ADE | -0.17 | - | 0.75 | 0.42 | - | - | - | 22,779.32 | 22,808.32 |
| *AE* | *0.19* | *-* | *0.81* | *-* | *3.010* | *1* | *.083* | *22,780.46* | *22,803.66* |
| CE | - | 0.11 | 0.90 | - | 7.013 | 1 | .008 | 22,785.38 | 22,808.59 |
| A=C | 0.07 | 0.07 | 0.86 | - | 5.293 | 1 | .021 | 22,783.22 | 22,806.72 |
| LDI |  |  |  |  |  |  |  |  |  |
| ACE | 0.32 | 0.13 | 0.56 | - | - | - | - | 8,322.56 | 8,353.04 |
| ADE | 0.70 | - | 0.56 | -0.26 | - | - | - | 8,322.56 | 8,353.04 |
| AE | 0.47 | - | 0.53 | - | 2.614 | 1 | .106 | 8,323.44 | 8,347.83 |
| CE | - | 0.35 | 0.65 | - | 8.290 | 1 | .004 | 8,330.75 | 8,355.14 |
| *A=C* | *0.21* | *0.21* | *0.58* | *-* | *1.015* | *1* | *.308* | *8,321.81* | *8,346.20* |

*Note.* Best fitting models for each variable are shown in italics. Tests of model fit are in reference to the full ACE model. SRH = Self-Rated Health; COMP = Comparative Health; ACT = Health Impact on Activities; a^2^  = additive genetic variance, c^2^ = shared environmental variance, d^2^ = dominant genetic variance, e^2^  = nonshared environmental variance, Adj = Adjusted for covariates age at baseline, sex, education, and depressive symptomatology; Δ*df* = difference in degrees of freedom between compared models; *p* = probability value; AIC = Akaike Information Criterion; BIC = Bayesian Information Criterion; A = additive genetic variance; E = nonshared environmental variance.

**Supplementary Table 2**

*Standardized Bivariate ACE Parameter Estimates of SH on LDI*

|  | SATSA | | | | LSADT | |
| --- | --- | --- | --- | --- | --- | --- |
| *Param* | SRH | SRH Adj. | ACT† | ACT Adj.† | SRH | SRH Adj. |
| β_A_ | -0.12 / [-.28, .03] | -.05/  [-.20, .01] | -.11* /  [-.20, -.01] | -.06 /  [-.15, .04] | -.30***/  [-.42, -.17] | -.16* /  [-.29, -.03] |
| β_E_ | - | - | - | - | -.12*** /  [-.19, -.06] | -.07* /  [-.13, -.004] |
| β_Age_ | -.20*** /  [-.28, -.12] | -.07 /  [-.18, .03] | -.20*** /  [-.28, -.11] | -.08 /  [-.18, .03] | -.14*** /  [-.17, -.11] | -.10*** /  [-.13, -.07] |
| β_Sex_ | - | .10* /  [.02, .19] | - | .10* /  [.02, .19] | - | .12*** /  [.09, .14] |
| β_Education_ | - | .16*** /  [.08, .23] | - | .16*** /  [.08, .23] | - | .19*** /  [.16, .22] |
| β_Dep._ | - | -.09* /  [-.17, -.01] | - | -.08* /  [-.16, -.002] | - | -.21*** /  [-.25, -.18] |
| β_Follow_ | - | .22*** /  [.10, .34] | - | .22*** /  [.10, .34] | - | .12*** /  [.08, .26] |

*Note.*  Estimates reported for best fitting models with and without adjustment for covariates. Covariates include age, sex, education, and depression. Age is included in all baseline models. SRH = Self-Rated Health; COMP = Comparative Health; ACT = Health Impact on Activities; Adj = Adjusted, β_A_= genetic effect of subjective health, β_E_= nonshared environment effect of subjective health, β_Age_ = effect of age, β_Sex_ = effect of sex, β_Education_ = effect of education, β_Dep._ = effect of depressive symptomatology, β_Follow_= effect of follow-up time.

† A and C SH variances indistinguishable in these models. b_A_ = represents effect of familial confounding

* p<.05, ** p<.01, *** p<.001

**Supplementary Table 3.**

*Goodness of Fit Statistics for Bivariate Twin Sex-Limitation Models*

| Model | *χ*^2^ | Δ*df* | *p* | AIC | BIC |
| --- | --- | --- | --- | --- | --- |
| **SATSA** |  |  |  |  |  |
| SRH |  |  |  |  |  |
| Baseline | - | - | - | 12,739.16 | 12,884.55 |
| *M = F* | *12.9* | *11* | *.300* | *12,731.11* | *12834.41* |
| COMP |  |  |  |  |  |
| Baseline | - | - | - | 12,731.48 | 12,876.87 |
| *M=F* | *13.03* | *11* | *.291* | *12,723.46* | *12,826.76* |
| ACT |  |  |  |  |  |
| Baseline | - | - | - | 12,619.92 | 12,765.30 |
| *M=F* | *12.25* | *11* | *.395* | *12,612.11* | *12,715.41* |
| **LSADT** |  |  |  |  |  |
| SRH |  |  |  |  |  |
| Baseline | - | - | - | 83,615.53 | 83,849.44 |
| M=F | 25.01 | 11 | .009 | 83,620.57 | 83,786.77 |
| *b_AM_=b_AF_, b_CM_=b_CF_, b_EM_=b_EF_* | *5.73* | *3* | *.126* | *83,618.62* | *83,834.072* |

*Note.* Best-fitting models shown in italics. Baseline model includes all ACE and regression parameters estimated uniquely for each sex. M = male, F = female, b_A_= genetic effect of subjective health, b_C_ = common environment effect of subjective health, b_E_= nonshared environment effect of subjective health,
